# Maize Elongin C interacts with the viral genome-linked protein, VPg, of *Sugarcane mosaic virus* and facilitates virus infection

**DOI:** 10.1111/nph.12890

**Published:** 2014-06-20

**Authors:** Min Zhu, Yuting Chen, Xin Shun Ding, Stephen L Webb, Tao Zhou, Richard S Nelson, Zaifeng Fan

**Affiliations:** 1 State Key Laboratory of Agro-biotechnology and Key Laboratory for Plant Pathology – Ministry of Agriculture, China Agricultural UniversityBeijing, 100193, China; 2 Plant Biology Division, The Samuel Roberts Noble Foundation, Inc.2510 Sam Noble Parkway, Ardmore, OK, 73401, USA; 3 Department of Computing Services, The Samuel Roberts Noble Foundation Inc.2510 Sam Noble Parkway, Ardmore, OK, 73401, USA

**Keywords:** *Brome mosaic virus*, eIF4E, Elongin C, maize (*Zea mays*), *Sugarcane mosaic virus* (SCMV), virus accumulation, virus-induced gene silencing (VIGS), viral genome-linked protein (VPg)

## Abstract

- The viral genome-linked protein, VPg, of potyviruses is involved in viral genome replication and translation. To determine host proteins that interact with *Sugarcane mosaic virus* (SCMV) VPg, a yeast two-hybrid screen was used and a maize (*Zea mays*) Elongin C (ZmElc) protein was identified.
- *ZmELC* transcript was observed in all maize organs, but most highly in leaves and pistil extracts, and ZmElc was present in the cytoplasm and nucleus of maize cells in the presence or absence of SCMV. *ZmELC* expression was increased in maize tissue at 4 and 6 d post SCMV inoculation. When *ZmELC* was transiently overexpressed in maize protoplasts the accumulation of SCMV RNA was approximately doubled compared with the amount of virus in control protoplasts.
- Silencing *ZmELC* expression using a *Brome mosaic virus*-based gene silencing vector (virus-induced gene silencing) did not influence maize plant growth and development, but did decrease RNA accumulation of two isolates of SCMV and host transcript encoding ZmeIF4E during SCMV infection. Interestingly, *Maize chlorotic mottle virus*, from outside the *Potyviridae*, was increased in accumulation after silencing *ZmELC* expression.
- Our results describe both the location of ZmElc expression in maize and a new activity associated with an Elc: support of potyvirus accumulation.

## Introduction

The members of the genus *Potyvirus* (family *Potyviridae*) cause significant yield and quality losses in a broad range of crop plants (Riechmann *et al*., 1992; Revers *et al*., 1999; Gibbs & Ohshima, 2010; Adams *et al*., 2012). Within grass species, the potyvirus *Sugarcane mosaic virus* (SCMV) is widespread and induces severe disease in maize (*Zea mays* L.), sugarcane (*Saccharum sinensis*) and sorghum (*Sorghum vulgare*) (Fuchs & Grüntzig, 1995; Shi *et al*., 2005; Użarowska *et al*., 2009). It is known as the major causal agent of maize dwarf mosaic disease in China, and the Beijing isolate (SCMV-BJ) belongs to a prevalent strain of SCMV in China (Fan *et al*., 2003). Yield losses can be as high as 30–50% and understanding the mechanism of infection by SCMV is critical for the identification of novel methods to control its accumulation and spread.

Potyviruses possess a single-stranded, positive-sense RNA genome *c*. 10 kb in length. A viral genome-linked protein, VPg, is attached to the 5′ terminus of the genomic RNA and a polyadenylate tract resides at the 3′ end of the genome. The viral genome can be translated to yield a polyprotein that is cleaved into ten mature proteins by three viral proteases (Urcuqui-Inchima *et al*., 2001). These proteins are responsible for virus accumulation and spread, suppression of RNA silencing and vector transmission (Urcuqui-Inchima *et al*., 2001; Adams *et al*., 2012). An additional protein, P3N-PIPO, resulting from a translation frame shift within the P3 cistron, was discovered and reported to influence virus cell-to-cell movement (Chung *et al*., 2008; Wei *et al*., 2010b; Vijayapalani *et al*., 2012). The continued analysis of the functions of these potyviral proteins is critical for exploring methods for virus control.

The VPg of potyviruses is covalently linked to the 5′ terminus of the genomic RNA via a tyrosine residue (Murphy *et al*., 1996; Anindya *et al*., 2005). VPg is an intrinsically disordered protein (Grzela *et al*., 2008; Rantalainen *et al*., 2008, 2011), and this property enables it to have multiple functions during virus infection (Rantalainen *et al*., 2008; Jiang & Laliberté, 2011). Potyviral VPg is a component of the virus replication complex and has been suggested to be the primer for negative-strand RNA synthesis because of its uridylylation, like the VPg of picornaviruses (Puustinen & Makinen, 2004). Other studies determined that VPg is involved in virus translation by either recruiting translation factors to promote viral RNA translation or sequestering translation factors to inhibit the formation of the translation initiation complex for host mRNAs (Léonard *et al*., 2000; Michon *et al*., 2006; Khan *et al*., 2008; Eskelin *et al*., 2011). VPg also influences potyvirus movement (Rajamaki & Valkonen, 2002; Dunoyer *et al*., 2004).

Several host proteins that interact with the VPg have been reported in the past decades. The best characterized is the eukaryotic translation initiation factor 4E (eIF4E) or its isoform, eIF(iso)4E. *Arabidopsis thaliana* eIF(iso)4E was the first identified VPg-interacting host protein (Wittmann *et al*., 1997). Later, a large number of VPg-eIF4E/eIF(iso)4E interactions were discovered from multiple hosts (Wang & Krishnaswamy, 2012). It is known that potyviral VPgs from some virus species selectively bind to specific isoforms of eIF4E (Lellis *et al*., 2002; Sato *et al*., 2005; Ruffel *et al*., 2006; Jenner *et al*., 2010). Studies showed that eIF4E or eIF(iso)4E was required for viral RNA translation (Khan *et al*., 2008; Miyoshi *et al*., 2008; Eskelin *et al*., 2011). Thus, knockout or mutation of either the *eIF4E* or *eIF(iso)4E* gene in the host can result in resistance to potyvirus infection (Duprat *et al*., 2002; Yeam *et al*., 2007; Charron *et al*., 2008; Rubio *et al*., 2009; Gallois *et al*., 2010; Hébrard *et al*., 2010; Ashby *et al*., 2011; Nieto *et al*., 2011). In addition to host translation proteins, cysteine-rich protein (Dunoyer *et al*., 2004), poly (A)-binding protein (PABP) (Léonard *et al*., 2004; Beauchemin & Laliberté, 2007; Dufresne *et al*., 2008), DEAD-box RNA helicase (AtRH8) and peach DDX-like protein (PpDDXL) (Huang *et al*., 2010), were identified as VPg interactors. Those interactions are reported to be crucial for virus infection and accumulation, although the underlying mechanisms for their actions remain unclear.

Elongin C was originally identified as a member of the mammalian transcription factor SIII that increases the rate of transcription by suppressing RNA polymerase II pausing (Bradsher *et al*., 1993a,b; Aso *et al*., 1995). As a central member of several multiprotein complexes, Elongin C is involved in a variety of activities including von Hippel-Lindau (VHL)-mediated tumor suppression (Duan *et al*., 1995; Yu *et al*., 2003) and cytokine signaling (Bullock *et al*., 2006; Babon *et al*., 2008) in mammalian cells. Other studies determined that it also acts as an E3 ligase within the ubiquitin-mediated proteolysis pathway in mammalian cells through binding with Elongin B (Gerber *et al*., 2004; Willems *et al*., 2004). In yeast, Elongin C is not involved in transcriptional stimulation (Koth *et al*., 2000). Yeast two-hybrid analysis demonstrated that yeast Elongin C interacts with a specific set of proteins involved in stress responses (Jackson *et al*., 2000). Yeast Elongin C, like its mammalian counterpart, is also known to be a component of E3 ligase complexes, in this situation influencing the DNA repair process (Ramsey *et al*., 2004; Gillette *et al*., 2006; Ribar *et al*., 2006, 2007; LeJeune *et al*., 2009). More recently, Elongin C was shown to participate in the spread of repressive histone modifications in *Chlamydomonas reinhardtii* (Yamasaki & Ohama, 2011). The only investigation of Elongin C in plants determined that *A. thaliana* Elongin C null mutants grew normally under experimental conditions, suggesting that it is dispensable for plant growth (Hua & Vierstra, 2011).

In this study, we identified a maize Elongin C (ZmElc) protein which interacts with SCMV VPg in both yeast and maize cells. We determined that the expression of *ZmELC* was induced in maize plant at 4 and 6 d post inoculation (dpi) with SCMV and ZmElc facilitated SCMV RNA accumulation in maize protoplasts when it was transiently overexpressed. By contrast, silencing its expression in maize plants through virus-induced gene silencing (VIGS) significantly reduced the accumulation of two different isolates of SCMV but increased the accumulation of *Maize chlorotic mottle virus* (MCMV), which is not within the *Potyviridae*. We also report that silencing *ZmELC* resulted in a decrease of *ZmeIF4E* expression in the presence of SCMV, although ZmElc did not interact directly with ZmeIF4E in our yeast or plant cell analyses.

## Materials and Methods

### Plasmid construction

Maize has two *ELC* members and the *ELC* we amplified is located on chromosome 6 (GenBank accession number: KJ811537) (determined through sequence analysis of the maize genome at Phytozome (http://www.phytozome.net/search.php)). Our primer-pairs used in this study were specific for the *ELC* identified in the yeast-two hybrid (Y2H) assay.

All of the constructs were sequenced before use. Information about the construction of all the plasmids is provided in Table 1. Sequences of all the primers used in this study are listed in Supporting Information Table S1.

**Table 1:** Construction of plasmids

Construct	Primer	PCR template
For Y2H assay (constructs were based on pGBKT7 and pGADT7 (Clontech))		
pGBKT7-SCMV-BJ VPg	VPg-1F, 1R	cDNA from virus infected maize tissue
pGBKT7-SCMV-OH VPg	VPg-1F, 1R	cDNA from virus infected maize tissue
pGBKT7-PenMV VPg	VPg-2F, 2R	cDNA from virus infected maize tissue
pGBKT7-TVBMV VPg	VPg-3F, 3R	cDNA from virus infected *Nicotiana benthamiana* tissue
pGBKT7-SCMV HC-Pro	Cheng *et al*. (2008)	
pGADT7-ZmELC	ELC-1F, 1R	maize cDNA
pGADT7-ZmeIF4E	eIF4E-1F, 1R	maize cDNA
pGADT7-ZmeIF(iso)4E	eIF(iso)4E-F, R	maize cDNA
For BiFC assay (constructs were based on pUC-SPYNE, pUC-SPYCE (Walter *et al*., 2004) and pGD (Goodin *et al*., 2002))		
SCMV-BJ VPg-YFP^N^	VPg-4F, 4R	cDNA from virus infected maize tissue
PenMV VPg-YFP^N^	VPg-5F, 5R	cDNA from virus infected maize tissue
TVBMV VPg-YFP^N^	VPg-6F, 6R	cDNA from virus infected *N. benthamiana* tissue
SCMV HC-Pro-YFP^N^	Cheng *et al*. (2008)	
pGD-SPYCE	YFP^C^-F, R	pUC-SPYCE
YFP^C^-ZmELC (pGD- YFP^C^-ZmELC)	ELC-2F, 2R	maize cDNA
ZmeIF4E-YFP^N^	eIF4E-2F, 2R	maize cDNA
ZmeIF4E-YFP^C^	eIF4E-2F, 2R	maize cDNA
For transient expression (constructs were based on pCAMBIA1390-GFP-N1, pCAMBIA1390-GFP-C1 (provided by Dr Elison Blancaflor) and pGD)		
pCAMBIA1390-GFP-ZmELC	ELC-3F, 3R	maize cDNA
pCAMBIA1390-ZmELC-GFP	ELC-4F, 4R	maize cDNA
pGD-GFP	GFP-1F, 1R	pCAMBIA1390-GFP-N1
pGD-GFP-ZmELC	ELC-2F, 2R	maize cDNA
For BMV-VIGS (constructs were based on a pC13/F3-13 m (Sun *et al*., 2013))		
pC13/F3-13 m: ELC	ELC-5F, 5R	maize cDNA
pC13/F3-13 m: GFP	GFP-2F, 2R	pCAMBIA1390-GFP-N1

### Virus and virus inoculations

SCMV-BJ and MCMV were from previously published sources (Fan *et al*., 2003; Zhang *et al*., 2011). Crude extracts were prepared by homogenizing the SCMV-BJ-, the Ohio isolate of SCMV- (SCMV-OH, provided by Dr Margaret G. Redinbaugh, Wooster, OH, USA) or MCMV-infected maize leaf tissues in 0.01 M phosphate buffer (pH 7.0) at 1 : 5 (w/v) ratio. The crude extracts were rub-inoculated individually to *Brome mosaic virus* (BMV)-inoculated maize leaves at 8, 5 or 6 dpi, respectively. The inoculated plants were again covered with plastic domes and grown inside a glasshouse set at 24°C.

### Yeast two-hybrid screen

The maize cDNA library screening was performed using a BD Matchmaker Library Construction and Screening Kit (Clontech, Mountain View, CA, USA) as instructed by the manufacturer. Positive colonies were isolated to obtain plasmid for sequencing and the sequences were analyzed through BLASTX searches.

### Particle bombardment

Particle bombardment was conducted as described (Finer *et al*., 1992), with the following specific modifications. To prepare a tungsten particle stock solution, 70 mg of tungsten particles (M17; Bio-Rad, Hercules, CA, USA) was mixed with 1.2 ml 75% ethanol in an Eppendorf tube by vortexing. After a 15-min incubation at room temperature, the tungsten particles were pelleted by centrifugation at 13 800 ***g*** for 5 min and the pellet was rinsed twice in RNase-free H_2_O. The pellet then was resuspended with 1.2 ml of 50% glycerol solution and stored at −80°C. For bombardment assays, 50 μl of the tungsten particle stock solution was mixed with 5 μl (*c*. 5 μg) plasmid DNA in an Eppendorf tube. After 5-min incubation on ice, 20 μl of 0.1 M spermidine solution was added to the tube followed by 10 μl of 2.5 M CaCl_2_ solution. The mixture was vortexed at low speed and then incubated at room temperature for 10 min. A 75% ethanol solution (700 μl) was added to the tube and the tungsten:plasmid DNA particles were pelleted at 17 000 ***g*** for 20 s. After two washes in 1 ml of 100% ethanol, the tungsten:plasmid DNA particles were pelleted again and then resuspended by pipetting in 400 μl of polyvinylpyrrolidone (PVP)/ethanol solution (1.6 μl PVP (20 mg PVP in 1 ml H_2_O) in 400 μl of ethanol). The tungsten:plasmid DNA solution was loaded into a Tefzel tube using a syringe. The tubing with the syringe attached was incubated for 10 min at room temperature. The PVP/ethanol solution was carefully withdrawn from the tubing and the tubing was allowed to dry inside a desiccator for 1 h. The dried tubing was cut into small pieces, inserted into individual slots inside a cartridge, and the tungsten:plasmid DNA particles were bombarded (220–250 psi) into maize leaves using the Helios Gene Gun system (Bio-Rad) as instructed.

### Preparation of MCMV RNA transcripts

The full-length cDNA clone of MCMV (pMCM41) was provided by Dr Kay Scheets (Stillwater, OK, USA). RNA transcripts of MCMV were synthesized as described (Scheets *et al*., 1993). The RNA transcripts were then treated with RNase-free DNase I (TaKaRa Bio Inc., Otsu, Japan) followed by two phenol/chloroform extractions and one chloroform extraction. The resulting supernatant was mixed with 3 M NaAc (pH 5.2) (10 : 1, v/v) and ethanol (1 : 2, v/v) followed by precipitation for 1 h at −80°C. The RNA transcripts were pelleted at 12 000 ***g*** for 5 min at 4°C and the pellet was washed twice with 1 ml 75% ethanol. The pellet was then resuspended in RNase-free double-distilled (dd)H_2_O.

### Maize protoplasts isolation and transfection

Protoplasts were isolated from leaves of maize inbred line Zheng 58 and transfected using a polyethylene glycol (PEG)-mediated method (Sheen, 1991) with modifications. Approximately 100 μl of maize protoplasts (1 × 10^5^) were gently mixed with 10 μg pGD-GFP or pGD-GFP-ZmELC, 5 μg of SCMV-BJ viral RNA from purified virus or MCMV RNA produced by *in vitro* transcription and 110 μl PEG/Ca solution (4 g PEG (MW 4000, Fluka), 2.5 ml 0.8 M mannitol, 1 ml 1 M CaCl_2_ or Ca(NO_3_)_2_ in 3 ml H_2_O) and incubated at room temperature for 15 min. The protoplasts were gently washed in 440 μl cold W5 solution (154 mM NaCl, 125 mM CaCl_2_, 5 mM KCl, 2 mM MES, pH 5.7) and then centrifuged at 150 ***g*** for 1 min. The pelleted protoplasts were resuspended in 1 ml W5 solution and incubated in the dark at 25°C. The transfected protoplasts were harvested at 12–18 h post transfection (hpt) and used for further analysis. Three independent experiments were conducted and 14 Eppendorf tubes with 1 × 10^5^ protoplasts each were used for each treatment within each experiment. Protoplasts in 10 tubes of the same treatment were pooled and used for protein extraction and Western blot assay as described previously (Cao *et al*., 2012). Protoplasts from the other four tubes were pooled for RNA isolation followed by qRT-PCR analysis.

### Confocal microscopy

For bimolecular fluorescence complementation (BiFC) assays and visualization of GFP in maize protoplasts and leaves, fluorescence signals were visualized under a Nikon Eclipse TE 2000-6 laser-scanning confocal microscope (Nikon, Tokyo, Japan). To visualize subcellular expression of GFP-ZmElc in maize cells, bombarded leaf tissue was examined under an UltraView ERS spinning-disc confocal microscope (Perkin-Elmer Life and Analytical Sciences, Turku, Finland) equipped with a × 63 water-immersion objective (Numerical aperture 1.40). The expressed YFP and GFP fusions were excited at 488 nm and GFP and YFP signal was captured at 522 nm. Images were processed using Adobe Photoshop (Adobe, San Jose, CA, USA).

### BMV-VIGS in maize

*Agrobacterium tumefaciens* cultures carrying pC13/F1 + 2, pC13/F3-13 m: ELC or pC13/F3-13 m: GFP were prepared as described (Sun *et al*., 2013). After induction, the *Agrobacterium* cultures were individually resuspended in an infiltration buffer (10 mM MES and 10 mM MgCl_2_) solution till OD_600_ = 2.0. An *Agrobacterium* culture harboring pC13/F1 + 2 was mixed with equal amount of an *Agrobacterium* culture harboring either pC13/F3-13 m: ELC or pC13/F3-13 m: GFP, and infiltrated into *Nicotiana benthamiana* leaves using needleless syringes. The 214-bp fragment of *ZmELC* (representing nucleotides 341–554 from the ATG start codon) amplified and inserted into the BMV vector was from the 3′-untranslated region of the gene which had 43.97% identity with the analogous region from the other maize *ELC*. In addition, there was no 21 nt or greater stretch of identity between the sequence inserted into the virus vector and the other maize *ELC*. Therefore, under accepted criteria for silencing (Burch-Smith *et al*., 2004), the BMV vector expressing our amplified *ELC* fragment would not directly induce silencing of the second maize *ELC* located on chromosome 9 (GenBank accession number: KJ811538).

At 3 dpi, BMV virions were isolated from the infiltrated *N. benthamiana* leaves as previously described (Lane, 1981) with the following modifications. The harvested leaf tissues were ground in liquid nitrogen and then homogenized in an extraction buffer (0.5 M NaAc and 0.8 M HAc) at a 1 : 2 ratio (w/v). The crude extract was loaded into 2 ml tubes, vortexed for 20 s, incubated on ice for 30 min and then centrifuged at 8000 ***g*** for 10 min at 4°C. The resulting supernatant was transferred into clean tubes and mixed (3 : 1, v/v) with 40% PEG (MW 8000, Sigma) containing 0.8 M NaCl followed by a 1-h incubation on ice with slow rocking. BMV virions were pelleted at 15 000 ***g*** for 15 min at 4°C and the pellets were resuspended in a small volume of 0.1 M phosphate buffer, pH 7.0. After 30 min incubation on ice, concentrations of partially purified BMV virions were estimated at OD_260_ using a BioPhotometer (Eppendorf, Hamburg, Germany).

BMV viral RNA was extracted from the virions as previously described (Dijkstra & de Jager, 1998) with the following specific modifications. Approximately 100 μl of partially purified BMV virions were mixed with 160 μl RNase-free ddH_2_O, 200 μl viral RNA extraction buffer (20 mM Tris-HCl, pH 8.0, 200 mM NaCl, 5 mM EDTA) and 40 μl 10% SDS, and incubated at room temperature for 5 min. After two phenol/chloroform extractions and one chloroform extraction, the resulting supernatant (*c*. 400 μl) was mixed with 3 M NaAc (pH 5.2) (10 : 1, v/v) and ethanol (1 : 2, v/v) followed by precipitation for 1 h at −80°C. Viral RNA was pelleted at 12 000 ***g*** for 10 min at 4°C and the pellet was washed twice with 1 ml 75% ethanol. Then the pellet was resuspended in 30 μl RNase-free ddH_2_O. Approximately 500 ng BMV viral RNA, the BMV-R primer (Table S1) and the M-MLV reverse transcriptase, was used to synthesize the first-strand cDNA as instructed (Promega). Two microliters of cDNA and primers BMV-1F and BMV-1R (Table S1) were used for PCR analyses to visualize the maintenance of foreign inserts in different BMV VIGS vectors.

Approximately 20 μg of partially purified BMV virions containing the full-length foreign inserts were rub-inoculated to each 1-wk-old Va35 maize seedling. The inoculated plants were covered with plastic domes and grown inside a growth chamber set at 18°C for 7 d before being transferred into a glasshouse set at 24°C with the domes removed.

### Total RNA extraction and qRT-PCR analysis of gene expression

Total RNA was extracted from maize tissue or protoplasts with TRIzol reagent (Invitrogen) and treated with RNase-free DNase I. First-strand cDNA was synthesized using 1 μg total RNA, an oligo (dT) primer or an MCMV-specific primer and M-MLV reverse transcriptase, as instructed. Ten-fold diluted cDNA product, gene-specific primers (Table S1) and the Power SYBR Green master mix (Applied Biosystems, Foster City, CA, USA) were used in PCR assays to quantitate SCMV RNA and *ZmELC* and *ZmeIF4E* transcript amounts with an ABI Prism 7900 HT sequence detection system (Applied Biosystems). *ZmEF1*α (elongation factor 1α) mRNA levels were determined with specific primers (Table S1) to allow normalization between transcript amounts among samples. The relative gene expression levels were calculated using the 2^−ΔΔ*C*T^ method (Livak & Schmittgen, 2001). The experiments were replicated at least three times.

## Results

### Identification of a VPg-interacting Elc from maize

**Fig. 1:**
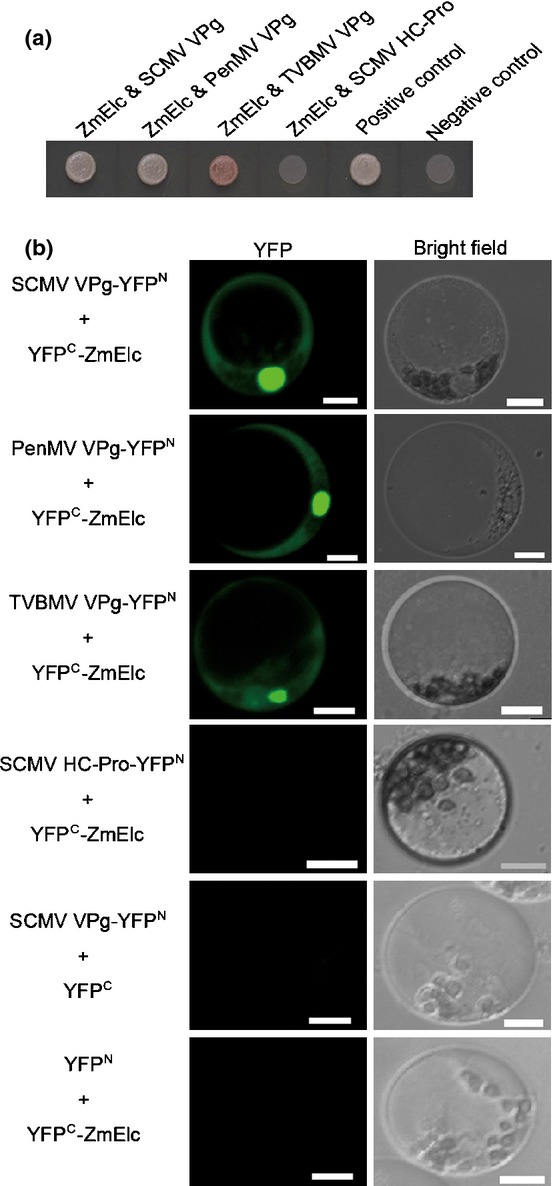
Interactions between ZmElc and viral genome-linked proteins (VPgs) from *Sugarcane mosaic virus* (SCMV), *Pennisetum mosaic virus* (PenMV) or *Tobacco vein banding mosaic virus* (TVBMV) and lack of interaction between ZmElc and SCMV HC-Pro in yeast and *in planta*. (a) Interactions between ZmElc and VPg of SCMV, PenMV or TVBMV and lack of interaction between ZmElc and SCMV HC-Pro in yeast. pGADT7-ZmELC and pGBKT7-SCMV-BJ VPg, pGBKT7-PenMV VPg, pGBKT7-TVBMV VPg or pGBKT7-SCMV HC-Pro were co-transformed into yeast strain AH109. The co-transformants were grown on the selective medium SD/-Ade/-His/-Leu/-Trp at 30°C for 3–4 d. (b) Interactions between ZmElc and VPg of SCMV, PenMV or TVBMV and lack of interaction between ZmElc and SCMV HC-Pro in maize protoplasts. Different combinations of BiFC vectors were co-transfected into maize protoplasts and YFP fluorescence signal was captured at 12–18 h post transfection. Protoplasts co-transfected with SCMV VPg-YFP^N^ and YFP^C^, and YFP^N^ and YFP^C^-ZmElc, were used as negative controls. Bars, 10 μm.

In order to identify maize proteins that interacted with SCMV-BJ VPg, a Y2H screen of a maize leaf cDNA library (Cheng *et al*., 2008) was performed using SCMV-BJ VPg as a bait. A total of 40 positive colonies were isolated for sequencing. One of them contained an intact ORF with 100% amino acid sequence identity to a maize Elc. The coding sequence of the Elc was cloned into pGADT7 and co-transformed with pGBKT7-VPg to yeast strain AH109. Using this cloned sequence in yeast the interaction between VPg and ZmElc in yeast was confirmed (Fig. 1a). To ensure that subsequent analyses were specific for this *ELC*, strictly specific primers were designed to amplify this *ELC* for transient expression and silencing studies and analysis of the *ELC* transcript amounts (see the Materials and Methods section).

In order to determine whether VPg and ZmElc interacted *in planta*, a BiFC assay was performed in maize protoplasts. The coding sequences of SCMV VPg and ZmElc were cloned into BiFC vectors pUC-SPYNE (YFP^N^) and pGD-SPYCE (YFP^C^) to generate SCMV VPg-YFP^N^ and YFP^C^-ZmElc, respectively. SCMV VPg-YFP^N^ and YFP^C^-ZmElc were co-transfected into protoplasts. Two other combinations of constructs, SCMV VPg-YFP^N^ and YFP^C^, and YFP^N^ and YFP^C^-ZmElc, were co-transfected into protoplasts as negative controls. YFP fluorescence was observed in both the nucleus and cytoplasm of the SCMV VPg-YFP^N^ and YFP^C^-ZmElc co-transfected protoplasts by 12–18 hpt (Fig. 1b). No YFP signal was detected in the negative controls (Fig. 1b). To determine the frequency of the interaction between SCMV VPg and ZmElc, we examined 100 protoplasts per experiment and calculated the numbers of the protoplasts exhibiting YFP signal from three experiments. The average percentage of protoplasts exhibiting fluorescence signal was 64 ± 4.5% (grand mean for three experiments ± SD). This result indicated that VPg and ZmElc interacted in maize cells.

In order to investigate the interaction specificity between ZmElc and VPgs, interactions between ZmElc and the VPgs from other members of the genus *Potyvirus*, *Pennisetum mosaic virus* (PenMV; Fan *et al*., 2004) and *Tobacco vein banding mosaic virus* (TVBMV; provided by Dr Xiangdong Li, Tai'an, Shandong), were analyzed through Y2H and BiFC assays. Positive interactions between ZmElc and both VPgs were observed in both assays (Fig. 1a,b). Y2H and BiFC assays also were used to determine whether ZmElc interacted with non-VPg proteins such as HC-Pro, P1 and CP of SCMV. No positive interaction was observed (Fig. 1a,b and data not shown).

### Expression analysis of *ZmELC*

**Fig. 2:**
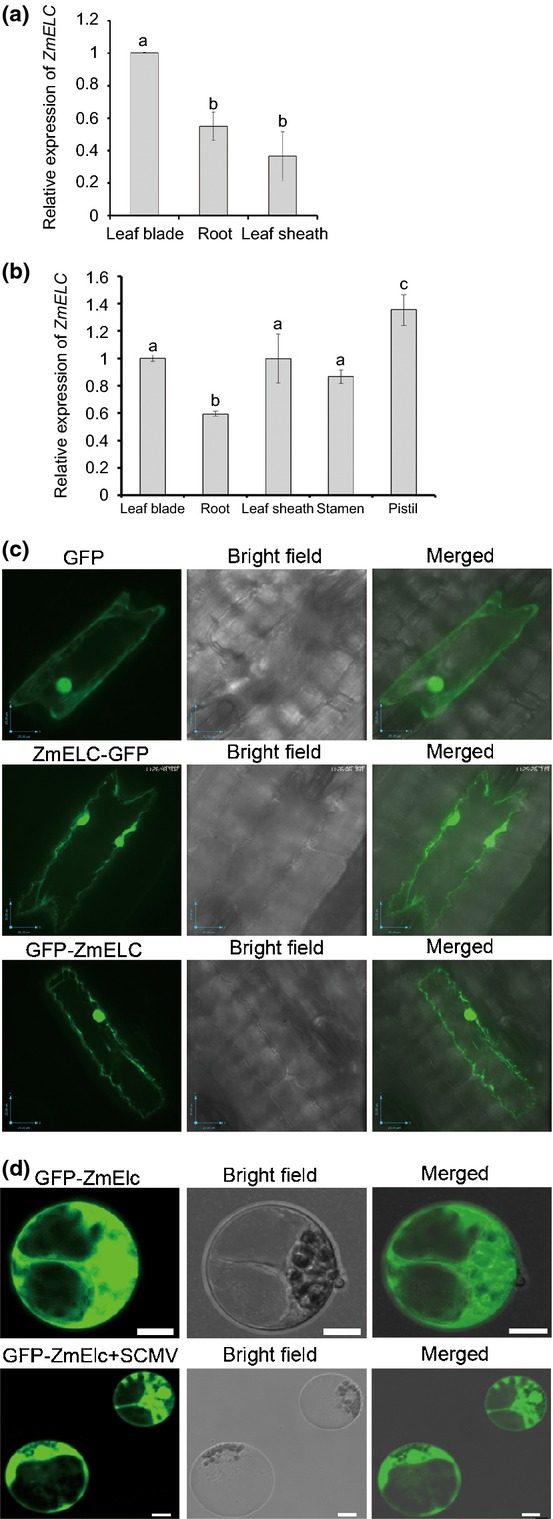
*ZmELC* expression in different maize organs and subcellular localization of ZmElc in maize protoplasts. (a) The expression of *ZmELC* in different organs of 14-d-old maize cv Va35. The transcript amount of *ZmELC* was determined through qRT-PCR using total RNA extracted from leaf blades, sheaths and roots. (b) The expression of *ZmELC* in different organs of adult maize cv Va35 plants (70 d post planting). A randomized complete block design (RCBD) analysis of variance (ANOVA) was used to determine if differences in the expression of *ZmELC* existed among organs for 14-d-old (a) or adult maize (b). Experiments were replicated three times; each independent experiment was used as the blocking factor in the RCBD ANOVA. When a significant *F*-test value was obtained, means separation tests using the Tukey–Kramer adjustment were performed. Bars represent the grand means ± SD, and different letters within an experiment indicate statistical significance at *P* ≤ 0.05. All analyses were carried out in SAS® 9.3 (SAS Institute, Inc (2011), Cary, NC, USA). (c) Subcellular location of ZmElc in maize cells. Tungsten:plasmid DNA particles were bombarded into maize cv Va35 leaves. The bombarded leaves were examined for the expression of fusion proteins at 36 h post bombardment. Bars, 25 μm. (d) Subcellular localization of ZmElc in maize protoplasts in the absence and presence of *Sugarcane mosaic virus* (SCMV)-BJ RNA. Maize protoplasts were transfected with pGD-GFP-ZmELC, or a mixture of pGD-GFP-ZmELC and SCMV-BJ RNA. GFP fluorescence signal was captured at 12–18 h post transfection. Bars, 10 μm.

In order to analyze whether *ZmELC* showed organ- or tissue-specific expression patterns, the relative amounts of *ZmELC* transcripts were determined by quantitative reverse transcription (RT)-PCR (qRT-PCR) using total RNA extracted from leaf blades, sheaths and roots of 14-d-old maize (cv Va35). The expression levels of *ZmELC* were similar in leaf sheath and root (Fig. 2a). However, the expression level of *ZmELC* was significantly higher in leaf blade than in leaf sheath and root (Fig. 2a).

A second analysis of *ZmELC* mRNA expression pattern was performed with total RNA extracts from both reproductive and vegetative organs of 70-d-old maize (cv Va35) plants. The *ZmELC* transcript quantity was highest in the pistil, with the stamen having a similar quantity to that in the leaf. The root had a lower quantity of *ZmELC* transcript than the other organs (Fig. 2b).

In order to study the subcellular localization of ZmElc in maize, leaves of maize (cv Va35) were bombarded with tungsten:plasmid DNA particles to express a fusion of ZmElc with GFP. The influence of the position of the fusion between ZmElc and GFP on subcellular location was evaluated by fusing ZmElc to either the N or C terminus of GFP (e.g. p1390-ZmELC-GFP and p1390-GFP-ZmELC) and then introduced into leaf cells through bombardment. Green fluorescence was observed at 36 h post bombardment. ZmElc distributed in both the cytoplasm and nucleus of the maize epidermal cells for both fusion proteins (Fig. 2c), suggesting that the location of the fusion, N or C terminus, had no effect on the subcellular location and was similar to that exhibited by free GFP (Fig. 2c). To understand the capacity for ZmElc to localize to the nucleus, a search at the NLSdb (https://rostlab.org/services/nlsdb), a database of nuclear localization signals (Nair *et al*., 2003), was performed and no nuclear localization signal was found within the ZmElc sequence.

In order to determine whether the nuclear localization of ZmElc was further enhanced during SCMV infection, presumably due to the known nuclear location of VPg (Rajamäki & Valkonen, 2009; Fig. 1b), maize protoplasts were transfected with pGD-GFP-ZmELC and SCMV-BJ RNA or pGD-GFP-ZmELC alone. Green fluorescence was observed at 12–18 hpt. ZmElc localized in both cytoplasm and the nucleus in maize protoplasts transfected with pGD-GFP-ZmELC alone (Fig. 2d), similar to the results obtained during its expression in uninfected maize epidermal cells (compare Fig. 2c with 2d). After co-transfection with SCMV-BJ RNA, the subcellular location of GFP-ZmELC was unchanged in the majority of protoplasts (> 99%, *n* > 60) (Fig. 2d).

### *ZmELC* expression was up-regulated at 4 and 6 d post SCMV inoculation

**Fig. 3:**
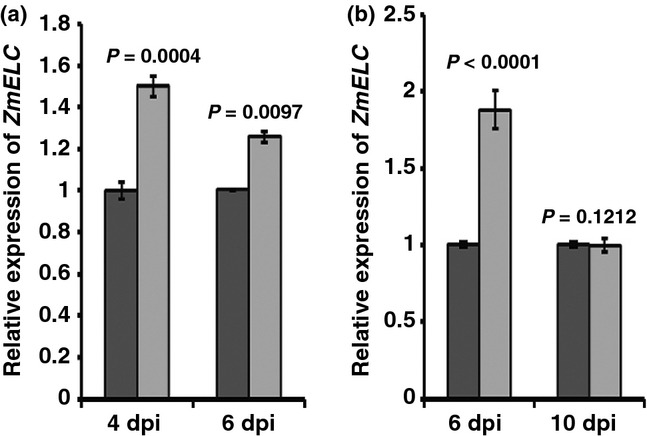
*ZmELC* expression is enhanced after *Sugarcane mosaic virus* (SCMV) infection. (a, b) Relative expression levels of *ZmELC* in SCMV- (light gray bars) and mock (dark gray bars)-inoculated maize leaves were determined at 4 and 6 d post inoculation (dpi) (a), and in the first systemically infected maize leaves at 6 and 10 dpi (b), all by qRT-PCR. Three independent experiments were conducted with four biological replicates each. Data were pooled across experiments and analyzed using a two-sample *t*-test. Bars represent the grand means ± SD. The *P* values are shown for each pair of treatments.

In order to determine whether *ZmELC* expression was induced by virus infection, 1-wk-old maize (cv Va35) seedlings were inoculated with SCMV-BJ or phosphate buffer (mock). Total RNA for qRT-PCR analysis was isolated from the inoculated leaves at 4 and 6 dpi, and from first systemically infected leaves at 6 and 10 dpi, respectively. *ZmELC* transcript amounts were *c*. 50% and 25% higher in virus-inoculated leaves than in mock-inoculated leaves at 4 and 6 dpi, respectively (Fig. 3a). The *ZmELC* transcript quantity was 87% higher in the first systemically infected leaves than in equivalent leaves from the mock-inoculated plants at 6 dpi (Fig. 3b). This difference disappeared at 10 dpi (Fig. 3b).

### Transient overexpression of *ZmELC* increased SCMV-BJ RNA accumulation in maize protoplasts

**Fig. 4:**
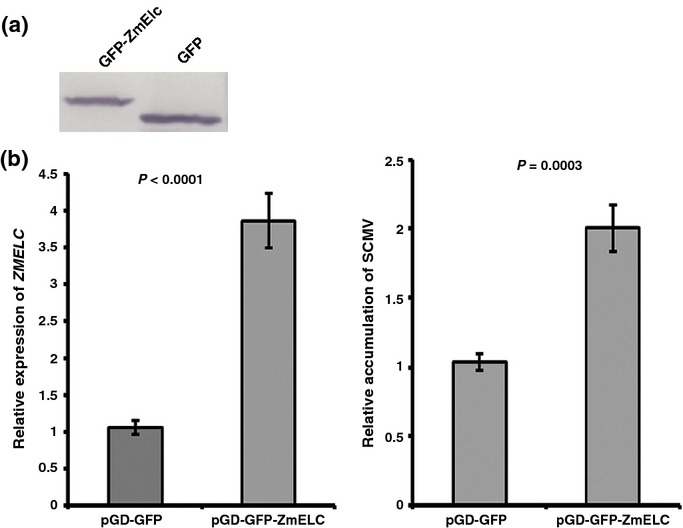
*Sugarcane mosaic virus* (SCMV)-BJ accumulation is increased during transient overexpression of *ZmELC* in maize protoplasts. Protoplasts were co-transfected with a mixture of pGD-GFP-ZmELC and SCMV-BJ RNA, or a mixture of pGD-GFP and SCMV-BJ RNA. The transfection rates of each treatment were determined at 18 h post transfection (hpt) by counting the number of protoplasts exhibiting green fluorescence in 100 protoplasts. Transfection rates for pGD-GFP and pGD-GFP-ZmElc treatments were 71.7% ± 2.9 and 71.0% ± 3.3, respectively. (a) Expression of GFP-ZmElc (left) and GFP (right) were determined by Western blot at 18 hpt. (b) The expression level of *ZmELC* (left) and the accumulation of SCMV RNA (right) in protoplasts co-transfected with pGD-GFP and SCMV-BJ RNA, and pGD-GFP-ZmELC and SCMV-BJ RNA at 18 hpt. Three independent experiments were conducted. Data were pooled across experiments and analyzed using a two-sample *t*-test. Bars represent the grand means ± SD. The *P* values are shown.

It is known that the VPg plays a key role in potyvirus replication. To investigate the possible role of ZmElc in SCMV replication, maize protoplasts were co-transfected with pGD-GFP-ZmELC and SCMV-BJ RNA. Protoplasts co-transfected with pGD-GFP and SCMV-BJ RNA were used as a control. GFP-ZmElc and GFP were expressed at 18 hpt as determined by Western blot using an anti-GFP antibody (Fig. 4a). qRT-PCR analyses were then conducted on RNA extracts at 18 hpt to quantify the relative expression levels of *ZmELC* transcript and SCMV-BJ RNA. When *ZmELC* transcript was overexpressed 3.9-fold, the SCMV-BJ RNA amount doubled compared with the control values (Fig. 4b). These findings indicated that transient overexpression of *ZmELC* was correlated with an increase in SCMV-BJ RNA accumulation.

In order to investigate whether transient overexpression of *ZmELC* influences the accumulation of another virus species in protoplasts, *in vitro* transcripts of MCMV, a member of the genus *Machlomovirus* (family *Tombusviridae*) and without a VPg, were co-transfected with either pGD-GFP or pGD-GFP-ZmELC into maize protoplasts. By 18 hpt, transient overexpression of *ZmELC* was correlated with significantly less accumulation of MCMV RNA (Fig. S1).

### Knockdown of *ZmELC* expression impaired SCMV infection in maize plants

**Fig. 5:**
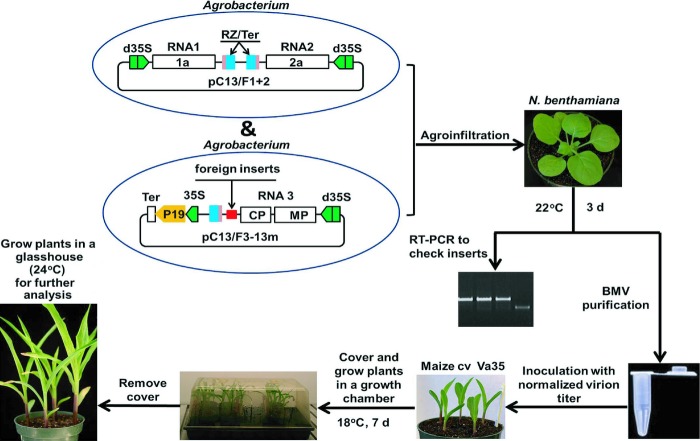
The modified method for virus-induced gene silencing (VIGS) in maize, using a DNA-based *Brome mosaic virus* (BMV) vector. For details, see the Materials and Methods section.

**Fig. 6:**
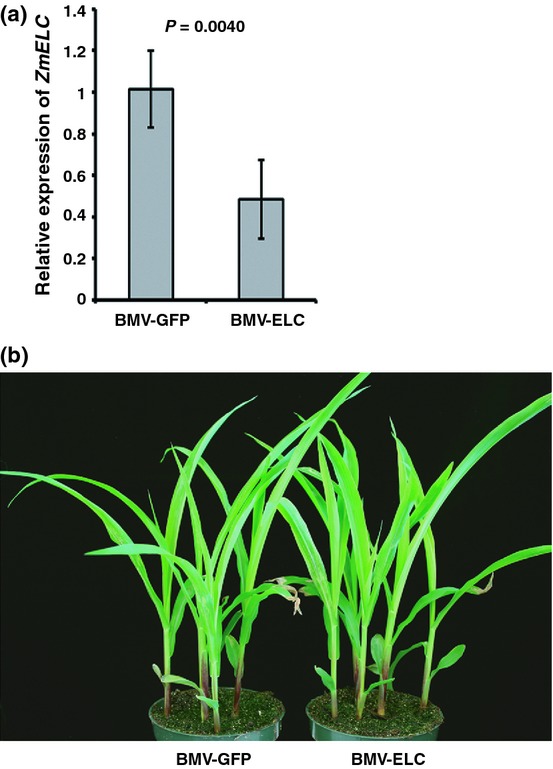
Knockdown of *ZmELC* using the *Brome mosaic virus* (BMV)-based virus-induced gene silencing (VIGS) vector. (a) Knockdown efficiency of *ZmELC* in the second systemically infected leaves at 13 d post inoculation (dpi). Three independent experiments were conducted with 11 biological replicates each. Data were pooled across experiments and analyzed using a two-sample *t*-test. Bars represent the grand means ± SD. The *P* value is shown. (b) BMV-GFP and BMV-ELC infected maize plants at 13 dpi.

In order to investigate further the role of ZmElc during SCMV infection, a newly modified DNA-based BMV VIGS vector (Benavente *et al*., 2012; Sun *et al*., 2013) was transformed into *A. tumefaciens* strain C58C1. *Agrobacterium* cultures harboring the virus vector containing a fragment of *ZmELC* were infiltrated into *N. benthamiana* leaves. VIGS vectors can lose foreign gene inserts over time post inoculation (Bruun-Rasmussen *et al*., 2007a) and this may be a major cause of the transient nature of the gene-silencing phenotype in grasses (Bruun-Rasmussen *et al*., 2007a; Ramanna *et al*., 2013). In addition, the virus without an insert accumulates to higher amounts than those still containing the insert (Bruun-Rasmussen *et al*., 2007a). To mimic the accumulation and stability characteristics of our BMV vector containing the *ZmELC* fragment, *Agrobacterium* harboring the BMV vector containing a 205-bp fragment of *GFP* gene was infiltrated into *N. benthamiana* leaves as a control. BMV virions containing full-length inserts were isolated from infiltrated *N. benthamiana* leaves at 3 d post agro-infiltration and rub-inoculated to leaves of 1-wk-old maize (cv Va35) seedlings (Fig. 5). Initial BMV infection symptoms were seen in the first systemically infected leaves by 5–7 dpi. In a preliminary experiment to determine the influence of BMV-GFP infection on expression of *ZmELC* in maize, it was determined that there was some inhibitory effect on *ZmELC* expression when compared with levels in mock-inoculated plants (Fig. S2). This further indicated the worth of the BMV-GFP vector as a control: to account for nonspecific effects of virus infection on *ZmELC* expression. In the study comparing *ZmELC* expression levels after inoculation with BMV-ZmELC and BMV-GFP, the second systemically infected leaves at 14–20 dpi were harvested from individually inoculated plants and analyzed for *ZmELC* silencing through qRT-PCR. *ZmELC* transcript amounts were decreased by *c*. 50% in BMV-ELC-inoculated plants compared with the amounts in BMV-GFP-inoculated plants (Fig. 6a). The plants silenced for *ZmELC* expression did not show any unusual visual phenotype compared with the BMV-GFP infected control plants (Fig. 6b).

**Fig. 7:**
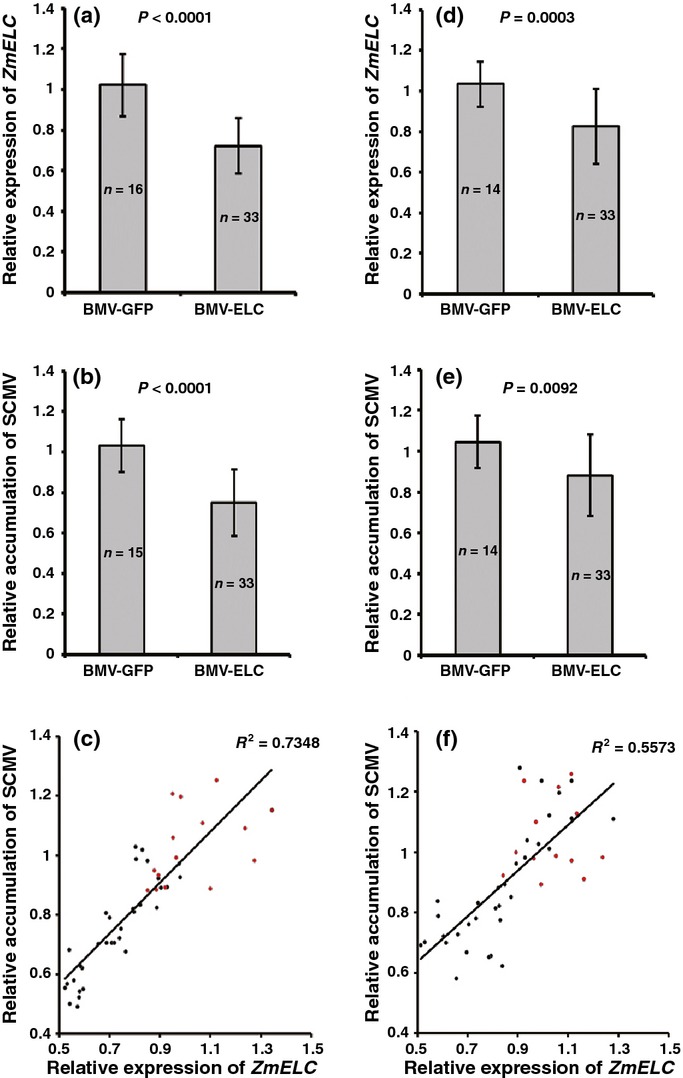
*Sugarcane mosaic virus* (SCMV)-BJ accumulation is decreased on knockdown of *ZmELC* expression in maize plants. (a, b) Knockdown efficiency of *ZmELC* expression (a) and relative SCMV-BJ RNA accumulation (b) at 5 d post inoculation (dpi) with SCMV in second systemically infected leaves. (d, e) Knockdown efficiency of *ZmELC* expression (d) and relative SCMV-BJ RNA accumulation (e) at 7 dpi with SCMV in second systemically infected leaves. A randomized complete block design (RCBD) analysis of variance (ANOVA) was used to determine the difference in *ZmELC* transcript and SCMV-BJ accumulation between *Brome mosaic virus* (BMV)-GFP and BMV-ELC inoculated plants at 5 dpi and 7 dpi. Experiments were replicated three times; each independent experiment was used as the blocking factor in the RCBD ANOVA. Bars represent the grand means ± SD. Sample sizes and the *P* values are shown. (c, f) Simple linear regression analyses were used to determine whether there was a relationship between expression of *ZmELC* (independent variable) and SCMV-BJ accumulation (dependent variable) at 5 dpi (c) and 7 dpi (f). Red dots represent plants inoculated with BMV-GFP and SCMV-BJ and black dots represent plants inoculated with BMV-ELC and SCMV-BJ. Regression analyses were significant at *P* < 0.001. All analyses were carried out in SAS® 9.3.

In the subsequent experiments, BMV-inoculated leaves were challenged with SCMV-BJ at 8 dpi. The second systemically infected leaf of each assayed plant was harvested at 5 and 7 dpi to determine *ZmELC* transcript knockdown efficiency and SCMV-BJ RNA accumulation. The results showed that at 5 dpi with SCMV, a 30% decrease in *ZmELC* mRNA levels (Fig. 7a) was associated with a 30% decrease in SCMV-BJ RNA accumulation (Fig. 7b). By 7 dpi, although the silencing of *ZmELC* in the BMV-ELC-inoculated plants was recovering (Fig. 7d), the inhibition of SCMV-BJ RNA accumulation was still significant (Fig. 7e). Simple linear regression analyses showed that there was a positive correlation between *ZmELC* expression and SCMV RNA accumulation at both 5 (Fig. 7c) and 7 dpi (Fig. 7f).

In order to determine if a similar relationship between *ZmELC* transcript amount and SCMV accumulation occurred across SCMV isolates, the relationship between *ZmELC* expression and the accumulation of the SCMV isolate, SCMV-OH, was analyzed. The interaction between SCMV-OH VPg and ZmElc was confirmed through the Y2H system (Fig. S3). As for the SCMV-BJ studies, silencing of *ZmELC* through VIGS resulted in decreased SCMV-OH accumulation, both effects decaying with time post inoculation (Fig. S3). Taken together, these results indicated that the knockdown of *ZmELC* was correlated with decreased accumulation of different isolates of SCMV.

In order to determine whether knockdown of *ZmELC* expression only inhibited accumulation of viruses encoding a VPg, leaves inoculated 6 d previously with the BMV silencing vectors were challenged with MCMV. Surprisingly, knockdown of *ZmELC* expression led to increased, rather than decreased, accumulation of MCMV at 7 dpi (Fig. S4).

### Knockdown of *ZmELC* expression in the presence of SCMV decreased *ZmeIF4E* expression

**Fig. 8:**
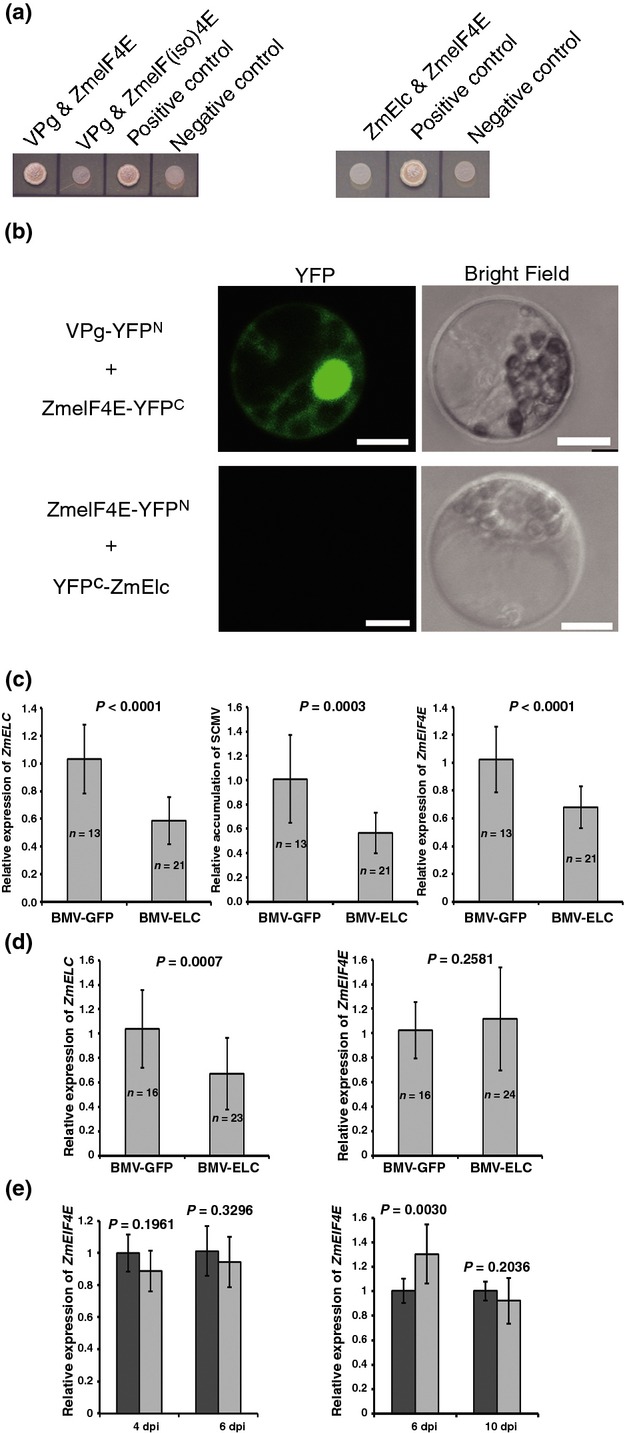
Interaction of *Sugarcane mosaic virus* (SCMV)-BJ viral genome-linked protein (VPg) with ZmeIF4E in yeast and maize cells, lack of interaction between ZmElc and ZmeIF4E in yeast and maize cells and downregulation of *ZmeIF4E* transcript amounts in plants silenced for *ZmELC* in the presence of SCMV-BJ. (a) The interactions between SCMV-BJ VPg and ZmeIF4E or ZmeIF(iso)4E (left) and lack of interaction between ZmeIF4E and ZmElc (right) in yeast. pGBKT7-SCMV-BJ VPg and pGADT7-ZmeIF4E or pGADT7-ZmeIF(iso)4E, and pGBKT7-ZmELC and pGADT7-ZmeIF4E were co-transformed into yeast strain AH109. The co-transformants were grown on the selective medium SD/-Ade/-His/-Leu/-Trp at 30°C for 3–4 d. (b) The interaction between SCMV-BJ VPg and ZmeIF4E (up), and lack of interaction between ZmeIF4E and ZmElc (down) in maize protoplasts. BiFC vectors, VPg-YFP^N^ and ZmeIF4E-YFP^C^, and ZmeIF4E-YFP^N^ and YFP^C^-ZmElc were co-transfected into maize protoplasts and YFP fluorescence signal was captured at 12–18 h post transfection. Bars, 10 μm. (c) *ZmELC* transcript (left), SCMV-BJ RNA (middle) and *ZmeIF4E* transcript (right) amounts in second systemically infected leaves at 5 d post inoculation (dpi) with SCMV-BJ. (d) *ZmELC* transcript amounts (left) and *ZmeIF4E* transcript amounts (right) in second systemically infected leaves at 13 dpi. A randomized complete block design (RCBD) analysis of variance (ANOVA) was used to determine the difference in *ZmELC* transcript, SCMV-BJ RNA and *ZmeIF4E* transcript amounts between *Brome mosaic virus* (BMV)-GFP and BMV-ELC inoculated plants. Experiments were replicated three times; each independent experiment was used as the blocking factor in the RCBD ANOVA. Bars represent the grand means ± SD. Sample sizes and the *P* values are also shown. All analyses were carried out in SAS^*®*^ 9.3. (e) Analysis of *ZmeIF4E* expression after SCMV infection. Relative expression levels of *ZmeIF4E* transcript in SCMV- (light gray bars) and mock (dark gray bars)-inoculated maize leaves were determined by qRT-PCR at 4 and 6 dpi (left), and in the first systemically infected maize leaves at 6 and 10 dpi (right). Three independent experiments were conducted with four biological replicates each. Data were pooled across experiments and analyzed using a two-sample *t*-test. Bars represent the grand means ± SD. The *P* values are shown for each pair of treatments.

It is well known that eIF4E or its isoform, eIF(iso)4E, is necessary for potyvirus accumulation and their absence provides recessive resistance to potyviruses in plants (Hébrard *et al*., 2010; Ashby *et al*., 2011; Mazier *et al*., 2011; Nieto *et al*., 2011). eIF4E belongs to a small multigenic family and two eIF4E isoforms, eIF4E and eIF(iso)4E, have been identified in maize (Manjunath *et al*., 1999). It was reported that potyviruses need only one specific eIF4E isoform to multiply in a specific host, although some potyviruses use both of them (Ruffel *et al*., 2006; Hwang *et al*., 2009). To identify which eIF4E isoform was needed by SCMV, a Y2H assay was performed and a positive interaction was found only between ZmeIF4E and VPg (Fig. 8a). This interaction was further confirmed by the BiFC assay in maize protoplasts (Fig. 8b). These results implied that ZmeIF4E may be employed by SCMV during its infection in maize.

In order to investigate if there is a relationship between the amounts of *ZmeIF4E* and *ZmELC* transcripts during SCMV infection, we inoculated maize plants using the same BMV-VIGS vector and SCMV-BJ inoculation procedure described above. The second systemically infected leaf of each assayed plant was harvested at 5 dpi to determine the *ZmELC* transcript knockdown efficiency, SCMV-BJ RNA accumulation and expression of *ZmeIF4E* transcript by qRT-PCR. The result indicated that, in the presence of SCMV and after knockdown of *ZmELC*, the expression of *ZmeIF4E* was decreased (Fig. 8c). To determine whether the decrease of *ZmeIF4E* expression also occurred in the absence of SCMV, developmentally similar leaves from plants infected only with the BMV-VIGS vector were sampled for qRT-PCR analysis at 13 dpi. Expression of *ZmeIF4E* transcript was not affected by the knockdown of *ZmELC* in the absence of SCMV (Fig. 8d). To exclude the possibility that the decreased expression of *ZmeIF4E* was due to SCMV infection, the *ZmeIF4E* transcript quantity was detected in the SCMV- and mock-inoculated leaves at 4 and 6 dpi, and the first systemically infected leaves of these plants at 6 and 10 dpi. The transcript quantity of *ZmeIF4E* in the SCMV-inoculated plants was similar to that in the mock-inoculated plants at most of these time points, although *ZmeIF4E* transcript quantity in first systemically infected leaf was *c*. 30% higher than that in the same leaf from the mock-inoculated plants at 6 dpi (Fig. 8e). This result indicated that SCMV infection alone did not downregulate *ZmeIF4E* expression. In addition, the Y2H and BiFC assay showed that there was no direct interaction between ZmElc and ZmeIF4E (Fig. 8a,b). Taken together, these data suggest that in the presence of SCMV, knockdown of *ZmELC* caused less *ZmeIF4E* expression.

## Discussion

Early studies on Elongin C showed that it was important for suppressing the pause by RNA polymerase II during the elongation phase of transcription in mammalian cells (Bradsher *et al*., 1993a,b). It later was determined that the elongin complex with Elongin C did not stimulate transcription elongation (Koth *et al*., 2000), but did play roles in stress responses by targeting specific factors that regulated protein kinase activities in yeast (Jackson *et al*., 2000). In mammalian cells it was demonstrated that this small protein worked as a core component of the Skp1-Cullin-F-box (SCF)-like ubiquitin ligase (E3 ligase) (Iwal *et al*., 1999). To date, the expression pattern and physiological function of Elongin C is not known in plants. In our study, we identified a ZmElc that interacted with SCMV VPg in both yeast and plant cells (Fig. 1a,b). The expression levels of *ZmELC* transcript were higher in the leaf blade than in the leaf sheath and root in 14-d-old maize seedlings (Fig. 2a). In adult maize plants the highest *ZmELC* transcript quantity was in the pistil (Fig. 2b). In maize cells, ZmElc located in both cytoplasm and nucleus (Fig. 2c,d). These findings provide the first evidence of Elongin C organ and subcellular localization in plants. Our observation that plants knocked down for *ZmELC* expression through VIGS did not show an abnormal visible phenotype (Fig. 6b) agreed with a previous report that Elongin C *Arabidopsis* null mutants were unaffected in growth (Hua & Vierstra, 2011).

The *ZmELC* we identified in this study is located in maize chromosome 6 and belongs to a maize two gene family. The other *ZmELC* is located in chromosome 9 and shares a low nucleotide sequence identity with chromosome 6 *ZmELC* in the 3′ untranslated region, although both *ZmELC*s have high nucleotide identity in the first 75% of the ORF from the start codon (Fig. S5a). All of our expression analyses were specific for the *ELC* encoded on chromosome 6 and thus, the lack of an altered visual phenotype in maize plants silenced for expression of this *ZmELC* may be due to the residual expression of this gene in our silenced plants and/or to functional complementation by the other member in this gene family. However, functional complementation by the *ZmELC* encoded in chromosome 9 might be minor, because its RNA expression level in leaf blades (at similar age and development to those used in our VIGS studies) was only 0.3–0.4% of the expression level of *ZmELC* from chromosome 6 (Fig. S5b).

Plant viruses have small genomes that encode a very limited number of proteins. Therefore, they depend on host factors to complete their infection cycles. As an important host factor involved in SCMV accumulation, ZmElc may be hijacked by the virus from its normal role in the plant to act as an enhancer during viral RNA replication. This speculation is supported by the fact that the ZmElc interacted with SCMV VPg, the suggested primer for viral RNA replication, and the accumulation of SCMV RNA was increased when *ZmELC* was transiently overexpressed in maize protoplasts (Fig. 4b). This may also explain why ZmElc was not further re-located into the nucleus by VPg through its interaction after SCMV infection (Fig. 2d): cytoplasmic localization is needed for potyvirus replication (Wei & Wang, 2008; Cotton *et al*., 2009; Laliberté & Sanfaçon, 2010; Wei *et al*., 2010a).

We also determined in this study that ZmElc interacted with the VPg from other potyviruses, PenMV and TVBMV (Fig. 1a,b). This suggests that these viruses may also require this protein for normal replication. However, the positive influence of Elongin C on virus accumulation may be limited to the potyviruses, because overexpressing or silencing *ZmELC* resulted in, respectively, reduced or enhanced accumulation of MCMV (Fig. S1, S4). In this instance, Elongin C may compete with MCMV proteins for host factors necessary for virus multiplication. It will be meaningful to carry out additional investigations to determine the role of Elongin C during the life cycles of different virus species, which, in turn, may further define the function of plant Elongin C in the absence of virus infection.

VPg is reported to interact with several proteins of both viral and host origin. Within the interaction network, the intrinsically disordered VPg acts as a hub protein that regulates many processes during virus infection (Jiang & Laliberté, 2011). Potyvirus VPg functions in viral RNA translation through its interaction with host eIF4E or its isoform, eIF(iso)4E. Host eIF4E binds to the 5′ cap structure of mRNA and it is best known for its essential function in the initiation of mRNA translation (Jackson *et al*., 2010). We determined that SCMV VPg bound ZmeIF4E in addition to ZmElc, but the two host proteins did not interact directly with each other (Fig. 8a,b). It is possible that ZmElc facilitates SCMV accumulation by interacting with the VPg during viral RNA replication before an interaction between VPg and ZmeIF4E, which is necessary for viral RNA translation. In this scenario, the downregulation of *ZmELC*, which was shown to inhibit SCMV RNA accumulation, would provide less viral RNA for translation and less requirement for ZmeIF4E and its transcript. Indeed, *ZmeIF4E* transcript amounts were decreased after silencing *ZmELC* and challenging with SCMV (Fig. 8c,d). It was shown that the expression of *Brassica perviridis* eIF4E protein can be induced by TuMV infection (Léonard *et al*., 2004). It is still unknown if *eIF4E* transcript amounts could be induced by potyvirus infection, but our evidence showed that it can be induced, at least transiently (Fig. 8e). Because *eIF4E* transcript can be induced during virus infection, it is possible that *ZmeIF4E* transcript amounts would decline when less virus accumulates due to *ZmELC* silencing.

Many recessive resistance genes against potyviruses have been identified in the last decade, such as *sbm-1* and *sbm-2* in pea (Johansen *et al*., 2001; Gao *et al*., 2004), *pvr 1*, *2* and *6* in pepper, (Ruffel *et al*., 2002; Kang *et al*., 2005), *mol*^*1*^ and *mol*^*2*^ in lettuce (Nicaise *et al*., 2003), *rym 4* in barley (Kanyuka *et al*., 2005) and *wlv* in white lupin (Bruun-Rasmussen *et al*., 2007b). Most of these genes encode eIF4E or its isoform eIF(iso)4E. In this study, we showed that ZmElc could facilitate virus accumulation; thus, maize mutants lacking ZmElc should be more resistant to virus infection. Future experiments should challenge the *Arabidopsis* Elongin C null mutants with a potyvirus to determine if they are more resistant. In addition, investigations should be made into whether knockdown of *ELONGIN C* can confer a broad resistance against other potyviruses in other plant species.

In the first report of VIGS in maize using the BMV-based vector, *in vitro* transcribed BMV RNAs were used to inoculate plants (Ding *et al*., 2006). In a later study the silencing vector was propagated in *N. benthamiana*, an intermediate host for BMV, before inoculation to the target grass plant (Ding *et al*., 2007). To obtain more uniform infection in various BMV-VIGS experiments, a modified inoculation procedure was established through normalizing virus titers between *N. benthamiana* extracts by qRT-PCR before inoculating maize leaves (van der Linde *et al*., 2011). In a more recent report a DNA-based BMV-VIGS vector was described and used to silence genes in rice and maize through *Agrobacterium*-mediated vacuum infiltration or vascular puncture inoculation (Benavente *et al*., 2012; Sun *et al*., 2013). However, the reported *Agrobacterium*-mediated vacuum infiltration was not successful in maize and therefore we further modified the inoculation method described by van der Linde *et al*. (2011) for maize. After propagating the virus in *N. benthamiana* leaves, virions were partially purified from the infiltrated leaves and virions maintaining full-length inserts were quantitated before inoculation to individual maize plants (Fig. 5). This modified inoculation method is easy to perform and ensures more uniform infection and gene silencing in maize. *Barley stripe mosaic virus* (BSMV) infection was reported to change the expression of common plant defense-related genes and resulted in a decreased susceptibility of wheat to *Magnaporthe oryzae* (Tufan *et al*., 2011). Although the effect of the BMV-VIGS vector on host defense-related genes remains unexplored, using a similar amount of BMV inocula in different treatments should equalize, and thus, minimize any confounding general effects caused by BMV infection alone during VIGS studies. In addition this new method allows the production of large amounts of recombinant BMV with full-length foreign inserts at a low cost.

## Supporting Information

**Fig. S1** MCMV accumulation is inhibited during transient overexpression of *ZmELC* in maize protoplasts. **Fig. S2** *ZmELC* expression was inhibited during BMV-GFP infection in maize leaves. **Fig. S3** SCMV-OH VPg and ZmElc interact in yeast and decreased SCMV-OH accumulation on knockdown of *ZmELC* expression in maize plants. **Fig. S4** MCMV accumulation is increased on knockdown of *ZmELC* expression in maize plants. **Fig. S5** Nucleotide sequence alignment and relative expression levels of the two *ELC* genes in maize. **Table S1** List of primers
