# Supplementary material for: Maize Elongin C interacts with the viral genome-linked protein, VPg, of Sugarcane mosaic virus and facilitates virus infection
Source: New Phytol. 2014 Jun 20;203(4):1291–304. doi: 10.1111/nph.12890 (PMC4143955; doi:10.1111/nph.12890)
Supplement: Supplementary file 1 [file nph0203-1291-SD1.pdf]

Supporting Information Figs S1-S5 and Table S1

Fig. S1

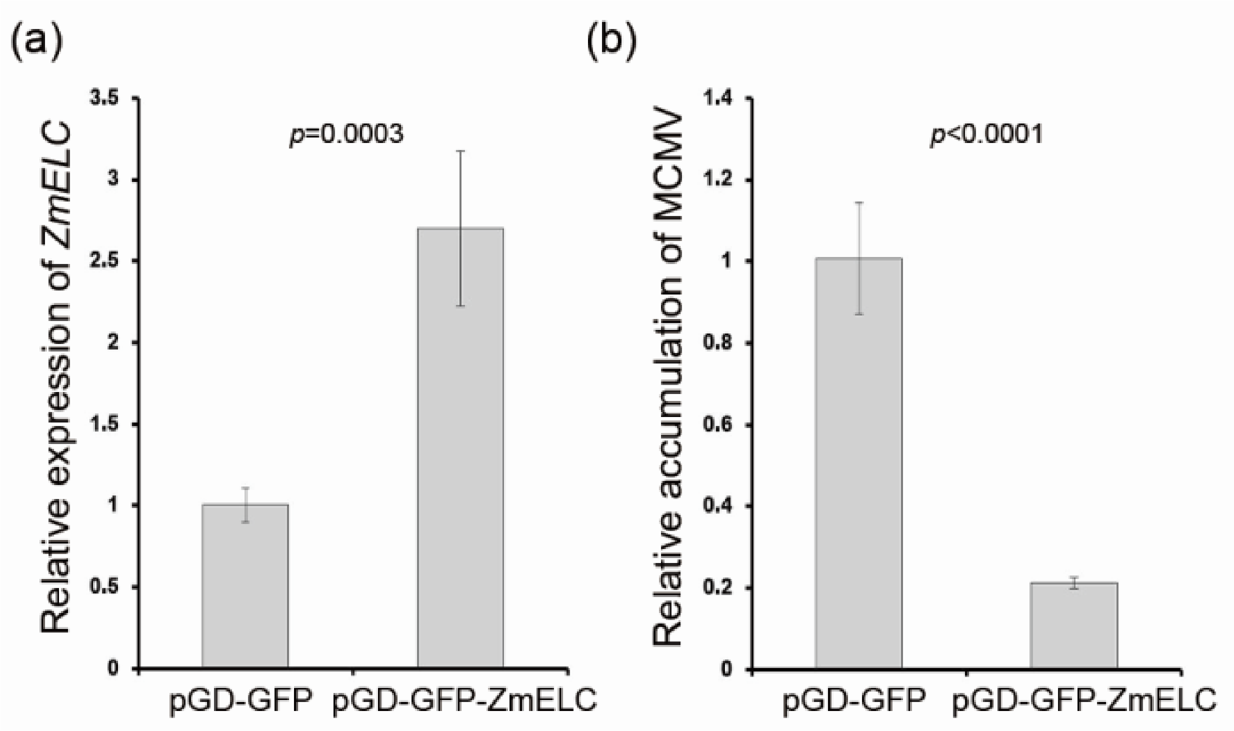

Fig. S1. MCMV accumulation is inhibited during transient overexpression of *ZmELC* in maize protoplasts. Protoplasts were co-transfected with a mixture of pGD-GFP-ZmELC and MCMV *in vitro* transcripts, or a mixture of pGD-GFP and MCMV *in vitro* transcripts. The expression level of *ZmELC* (a) and the accumulation of MCMV RNA (b) in these protoplasts at 18 hours post transfection are shown. Three independent experiments were conducted. Data were pooled across experiments and analyzed using a two-sample *t*-test. Bars represent the grand means ± standard deviations. The *p* values are shown.

Fig. S2

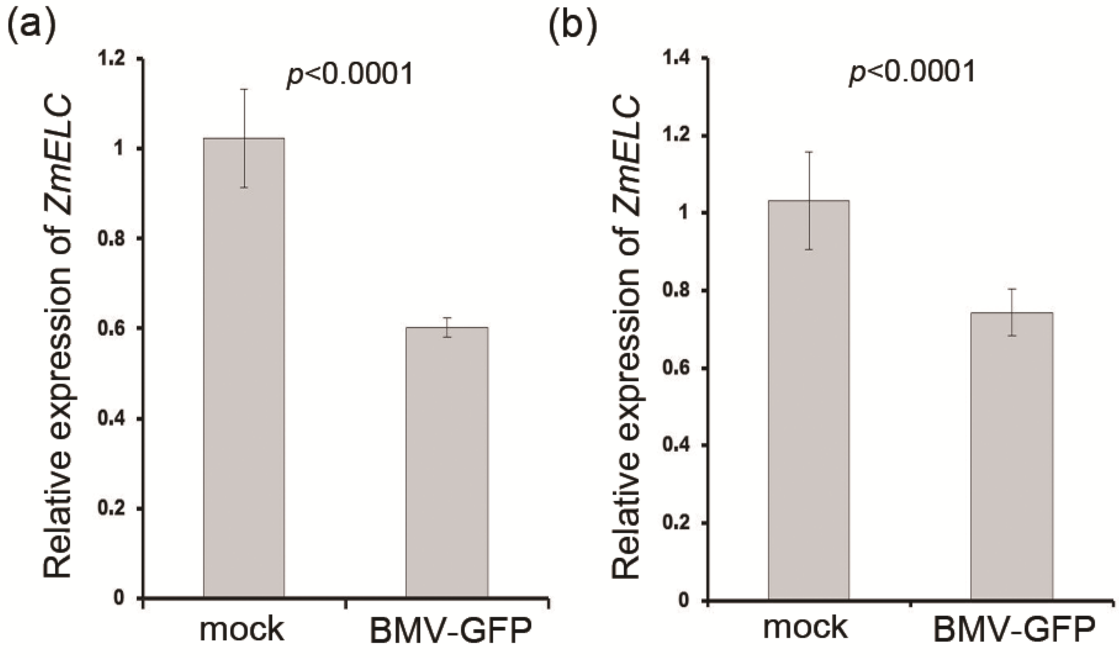

Fig. S2. *ZmELC* expression was inhibited during BMV-GFP infection in maize leaves. Relative expression levels of *ZmELC* in the second-systemically infected leaves of BMV-GFP- and mock-inoculated plants were determined at 13 (a) and 15 dpi (b) by qRT-PCR. Three independent experiments were conducted with four biological replicates per treatment. Data were pooled across experiments and analyzed using a two-sample *t*-test. Bars represent the grand means ± standard deviation for each treatment. The *p* values are shown.

Fig. S3

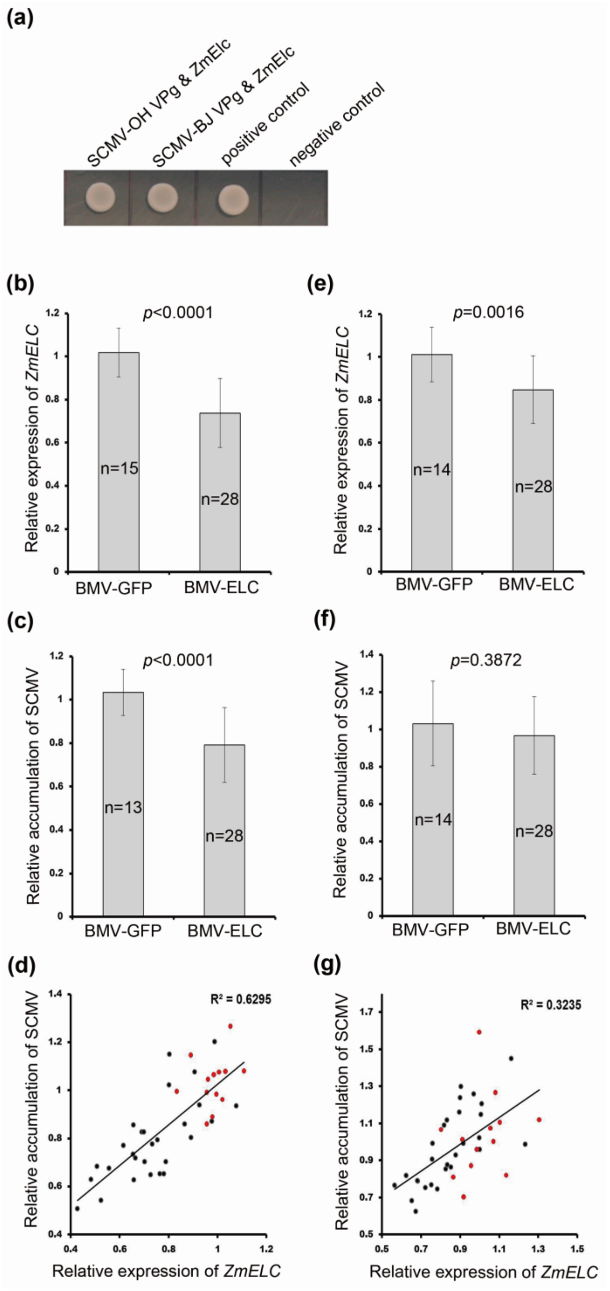

Fig. S3. SCMV-OH VPg and ZmElc interact in yeast and decreased SCMV-OH accumulation on knockdown of *ZmELC* expression in maize plants. (a) Interaction between SCMV-OH VPg and ZmElc in yeast. pGBKT7-SCMV-OH VPg and pGADT7-ZmELC were co-transformed into yeast strain AH109. The co-transformants were grown

on the selective medium SD/-Ade/-His/-Leu/-Trp at 30°C for 3-4 days. (b, c) Knockdown efficiency of *ZmELC* expression (b) and relative SCMV-OH RNA accumulation (c) at 8 days post SCMV inoculation in second-systemically infected leaves. (e, f) Knockdown efficiency of *ZmELC* expression (e) and relative SCMV-OH RNA accumulation (f) at 10 days post SCMV inoculation in second-systemically infected leaves. A randomized complete block design was used and the experiment was repeated three times. To determine the difference in *ZmELC* transcript and SCMV-OH accumulation between BMV-GFP and BMV-ELC inoculated plants at 8 dpi and 10 dpi, an analysis of variance (ANOVA) using general linear mixed models was conducted. Each independent experiment (n=3) was used as the blocking factor in the ANOVA. Data were adjusted for unequal variance between treatments and by using the Satterthwaite denominator degrees of freedom adjustment for the unequal variances (SAS Institute, Inc., 2011). Bars represent the grand means ± standard deviations. Sample sizes and the p values are also shown. (d, g) Simple linear regression analyses were used to determine whether there was a relationship between expression of *ZmELC* (independent variable) and SCMV-OH accumulation (dependent variable) at 8 dpi (d) and 10 dpi (g). Red dots represent plants inoculated with BMV-GFP and SCMV-OH and black dots represent plants inoculated with BMV-ELC and SCMV-OH. Regression analyses were significant at $P < 0.001$. All analyses were carried out in SAS® 9.3.

Fig. S4

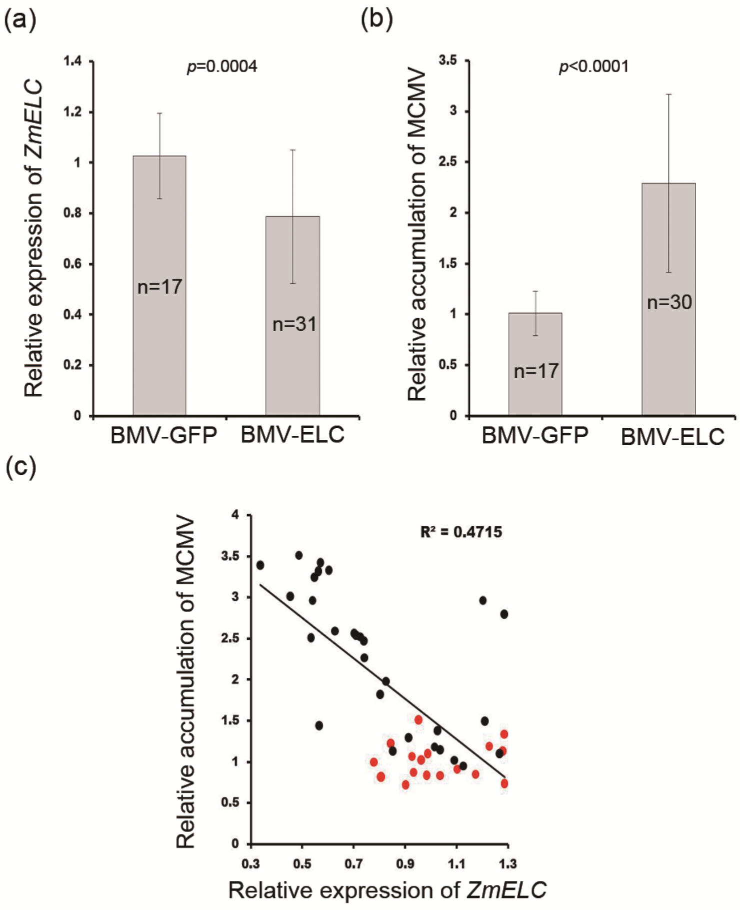

Fig. S4. MCMV accumulation is increased on knockdown of *ZmELC* expression in maize plants. Knockdown efficiency of *ZmELC* expression (a) and relative MCMV RNA accumulation (b) at 7 days post MCMV inoculation in second-systemically infected leaves. A randomized complete block design (RCBD) analysis of variance (ANOVA) was used to determine the difference in *ZmELC* transcript and MCMV accumulation between BMV-GFP and BMV-ELC inoculated plants at 7 dpi. ANOVAs were conducted using general linear mixed models where each independent experiment (n=3) was considered the blocking factor. Data were adjusted for unequal variance between treatments and by using the Satterthwaite denominator degrees of freedom adjustment for

the unequal variances (SAS Institute, Inc., 2011). Bars represent the grand means ± standard deviations. Sample sizes and the *p* values are shown. Simple linear regression analyses were used to determine whether there was a relationship between expression of *ZmELC* (independent variable) and MCMV accumulation (dependent variable) at 7 dpi (c). Red dots represent plants inoculated with BMV-GFP and MCMV and black dots represent plants inoculated with BMV-ELC and MCMV. Regression analyses were significant at $P < 0.001$. All analyses were carried out in SAS® 9.3.

Fig. S5

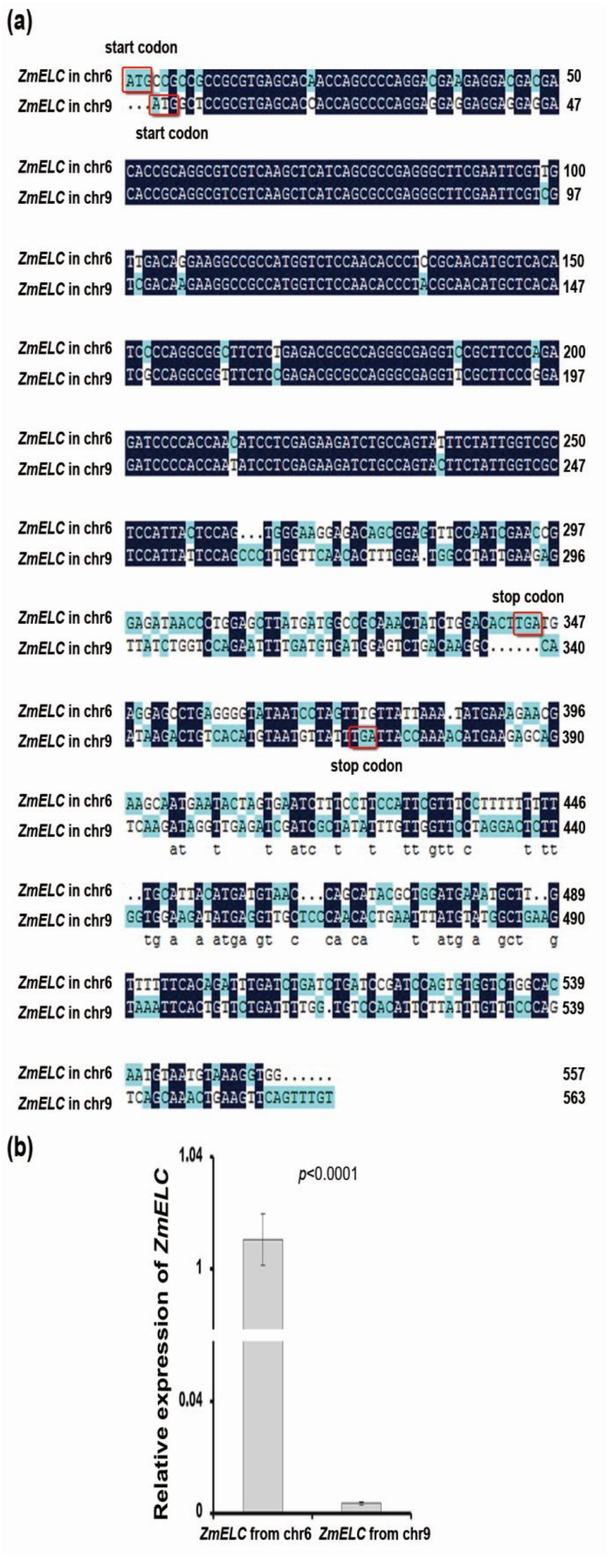
(a)
start codon
ZmELC in chr6
ZmELC in chr9
start codon
50
47
100
97
150
147
200
197
250
247
297
296
stop codon
347
340
396
390
stop codon
446
440
489
490
539
539
557
563
(b)
1.04
1
0.04
0
p<0.0001
Relative expression of ZmELC
ZmELC from chr6
ZmELC from chr9

Fig. S5. Nucleotide sequence alignment and relative expression levels of the two *ELC*s in maize. (a) Nucleotide sequence alignment of *ZmELC*s from chromosome 6 and 9. The gene coding region and part of 3' untranslated region of *ZmELC* in chromosome 9 was amplified by PCR using cDNA from 14-day-old maize cv. Va35 seedlings and primers ELC-6F and ELC-6R. The PCR products were cloned into the pGEM-T Easy vector (Promega Corp., Madison, WI) for sequencing. Start and stop codons of the two *ELC*s were shown. (b) Relative expression of *ZmELC*s from chromosomes 6 and 9 in leaf blades of 14-day-old maize cv. Va35 plants (representing the developmental stage similar to leaves sampled for VIGS studies). The transcript level of the two *ZmELC* was determined through qRT-PCR. First-strand cDNA was synthesized using 5 μg total RNA extracted from leaf blade and cDNA without dilution was used for qRT-PCR analyses. Two independent experiments were conducted with four biological replicates each. Data were pooled across experiments and analyzed using a two-sample *t*-test. Bars represent the grand means ± standard deviations. The *P* value of the t-test is shown on the figure.

Table S1. List of primers

Primers	Sequences (5'-3')
ELC-1F	TAGGATCCATATGCCGCCGCCGCGTGA (*Bam*HI)
ELC-1R	GCGAGCTCGTCAAGTGTCCAGATAGTTTGC (*Sac*I)
ELC-2F	ATAAAGCTTCGATGCCGCCGCCGCGTGAGC (*Hin*dIII)
ELC-2R	CGCGGATCCTCAAGTGTCCAGATAGTTTGCGG (*Bam*HI)
ELC-3F	ATAACTAGTATGCCGCCGCCGCGTGAGC (*Spe*I)
ELC-3R	GCGGGTGACCTGTCAAGTGTCCAGATAGTTTGC (*Bst*EII)
ELC-4F	AATGTCGACGATGCCGCCGCCGCGTGAGC (*Sal*I)
ELC-4R	ATCCCCGGGGAGTGTCCAGATAGTTTGCGGCC (*Sma*I)
ELC-5F	AGGCTTCCTAGGCTTGATGAGGAGCCTGAG (*Avr*II)
ELC-5R	AGGCTTCCATGGCCTTTACATTACATTGTGCC (*Nco*I)
ELC-6F	TCCGTTGCAGGTCGCAGGATG
ELC-6R	CACAAACTGAACTTCAGTTTGCTGAC
VPg-1F	TAACCATGGAGATGGGAAGAGACGAGCGCAGT (*Nco*I)
VPg-1R	TAACTGCAGGTTCATGTGCCACCCCCGCTT (*Pst*I)
VPg-2F	CTCGAATTCATGGGCAGATCAAAGAGAAG (*Eco*RI)
VPg-2R	GTAGGATCCCTCATTCATGTGTCACCCCTT (*Bam*HI)
VPg-3F	CCTCCATGGAGATGGGAAAGAACAAGAAAGGAGC (*Nco*I)
VPg-3R	AATGGATCCCTCACTCGTGATCAACTCCCTCAACC (*Bam*HI)
VPg-4F	CACACTAGTATGGGAAAGAACAAGCGCA (*Spe*I)
VPg-4R	AACCTCGAGTTCATGTGCCACCCCC (*Xho*I)
VPg-5F	CGGTCTAGAATGGGCAGATCAAAGAGAAGTAA (*Xba*I)
VPg-5R	TATGGATCCTTCATGTGTCACCCCTTCCT (*Bam*HI)
VPg-6F	GCGTCTAGAATGGGAAAGAACAAGAAAGGAG (*Xba*I)
VPg-6R	TTAGGATCCCTCGTGATCAACTCCCTCA (*Bam*HI)
eIF4E-1F	ATACATATGATGGCCGAGGAGACCGACAC (*Nde*I)
eIF4E-1R	ATAGGATCCCTCAAACCGTGTAACGTTCC (*Bam*HI)
eIF4E-2F	ATATCTAGAATGGCCGAGGAGACCGACAC (*Xba*I)
eIF4E-2R	ATACCCGGGAACCGTGTAACGTTCCTTCAG (*Sma*I)
eIF(iso)4E-F	ATAGAATTCATGGCGGAGGTCGAGGCTC (*Eco*RI)
eIF(iso)4E-R	TTAATCGATGTTACACGGTGTACCGCCCAC (*Cla*I)
GFP-1F	ATA*AGATCT*ATGGTGAGCAAGGGCGAGGAGC (*Bgl*II)
GFP-1R	CTTAAGCTTGCTTGTACAGCTCGTCCATGCC (*Hin*dIII)
GFP-2F	TGCTAATCCTAGGACATCACGGCAGACAAAC (*Avr*II)
GFP-2R	TGCTATCCATGGACCATGTGGTCACGCTTT (*Nco*I)
YFP^{C}-F	ATAAGATCTATGGACAAGCAGAAGAACGGCATC (*Bgl*II)
YFP^{C}-R	TTAAAGCTTGCTTGTACAGCTCGTCCATG (*Hin*dIII)
BMV-R	TGGTCTCTTTTAGAG
BMV-1F	CTTGTGTTGCTGAGAAAC
BMV-1R	TCTTGTAAGAGGTCTGC
**For reverse transcription of MCMV RNA**	
MCMV-RT	TCAATGATTTGCCAGCCCTGGGCCTGGAACCAGG
**For qRT-PCR**	
ELC6-F	ATTGGTCGCTCCATTACTCC
ELC6-R	CCATCATAAGCTCCAGGGTT

Primers	Sequences (5'-3')
ELC9-F	TCCATTATTCCAGCCCTTGGTTC
ELC9-R	GACAGTCTTATTGGCCTTGTCAG
SCMV-BJ-F	GGCGAGACTCAGGAGAATACA
SCMV-BJ-R	ACACGCTACACCAGAAGACACT
SCMV-OH-F	GGCGAGACCCAGGAGAATACA
SCMV-OH-R	CTATAGGTACTGCAAACAGGGTTTCC
eIF4E-F	GACAGGGAGGAGGGCGAGAT
eIF4E-R	GTGTATTGGGCGGATGGAGC
MCMV-F	GCATCACTTGGGAGACATTCAC
MCMV-R	CGGTTCTGTGGCATGACAAT
EF1α-F	GCCACACCTCACACATTGCT
EF1α-R	GCCGGATCGCCTATCAATCT
